# Tomato Varieties Influence the Performance of *Tamarixia triozae* (Hymenoptera: Eulophidae) on *Bactericera cockerelli* (Hemiptera: Triozidae) Nymphs

**DOI:** 10.3390/insects13090825

**Published:** 2022-09-11

**Authors:** Juan Mayo-Hernández, Jorge Luis Vega-Chávez, Agustín Hernández-Juárez, Yolanda Rodríguez-Pagaza, José Humberto Valenzuela-Soto, Alberto Flores-Olivas

**Affiliations:** 1 Departamento de Parasitología, Universidad Autónoma Agraria Antonio Narro, Buenavista, Saltillo C.P. 25315, Coahuila, Mexico; 2 Instituto Tecnológico Superior de Huichapan, El Saucillo, Huichapan C.P. 42411, Hidalgo, Mexico; 3 CONACYT-Centro de Investigación en Química Aplicada. Blvd. Enrique Reyna No. 140, Col. San José de los Cerritos, Saltillo C.P. 25294, Coahuila, Mexico

**Keywords:** parasitism, *Tamarixia triozae*, *Bactericera cockerelli* nymphs, tomato varieties

## Abstract

**Simple Summary:**

Horticultural crops are often exposed to insect attacks, and insect vectors of plant diseases represent a significant agronomical challenge. The tomato psyllid is a principal vector for bacterial pathogens causing disease and economic losses in crops. In the present study, we evaluated the performance of the parasitoid (*T. triozae*) against tomato psyllid on tomato varieties. Our results showed that parasitoids selected one commercial variety of tomato plants and attacked the psyllid under greenhouse conditions. We also demonstrated that healthy plants of commercial variety attracted more natural enemies under laboratory conditions. Therefore, these results could help fine-tune the release of natural enemies in greenhouse and field crops.

**Abstract:**

The potato/tomato psyllid *Bactericera cockerelli* is the *Candidatus* Liberibacter solanacearum bacterium vector that causes diseases in Solanaceae crops. Pest control is based on synthetic chemical insecticides, plant extracts, and natural enemies such as parasitoids. *Tamarixia triozae* feeds on nymphs of *B. cockerelli*, reaching up to 95% parasitism. This work aimed to evaluate the parasitic performance of *T. triozae* on tomato leaves with *B. cockerelli* N3 nymphs, using two domesticated (Floradade and Micro-Tom) and one Wild tomato variety. Several assays were completed to identify the parasitoid attraction toward un-infested plants (healthy) and infested plants (damaged) of three varieties. Parasitism preference and “Y” tube olfactometer tests were performed, respectively. The parasitism of *Tamarixia triozae* showed a preference toward plants of the Floradade variety by 44% compared with the other two varieties (*p* = 0.0003). *T. triozae* was more attracted to damaged plants of the Wild variety (*p =* 0.0523). Healthy plants of Floradade and Micro-Tom varieties attracted a higher proportion of parasitoids, except in the Wild variety, where *T. triozae* was more attracted to damaged plants. Taken together, the results of this study show that the domestication degree in tomato plants positively influenced the interactions between tomato plants and the parasitoid, *T. triozae.*

## 1. Introduction

Throughout America’s history, agricultural crops have been attacked by many hemipteran insect vectors of plant pathogens such as aphids, thrips, whiteflies, and psyllids [1,2,3,4,5]. Among these, because of its direct feeding damage and ability to vector bacterial pathogen *Candidatus* Liberibacter in Solanaceae crops, the tomato/potato psyllid *Bactericera cockerelli* (Sulc) (Hemiptera: Triozidae) has become a key pest in multiple economically important crops such as potato (*Solanum tuberosum* L.), tomato (*Solanum lycopersicum* L.), pepper (*Capsicum annuum* L.), and eggplant (*Solanum melongena* L.) [6,7,8]. Because of this bacterium, between 2006 and 2008, yield losses of more than 20% were reported, equivalent to USD 33.4 million per year [9,10,11]. Its control is mainly based on synthetic chemical insecticides and plant extracts, which are primarily used as repellents [12,13,14]. For biological control of *B. cockerelli*, several natural enemies have been reported, among which the parasitoid *Tamarixia triozae* (Burks) (Hymenoptera: Eulophidae) stands out [15,16]. *Tamarixia triozae*’s potential for potato/tomato psyllid regulation has recently been studied [17]. *Tamarixia triozae* is a crucial ectoparasitoid that feeds on nymphs of the third, fourth, and fifth stages of *B. cockerelli* [16]. This feature gives it an advantage as a biological control agent by eliminating the psyllid nymphs via oviposition and predation [18]. Depending on the crop management conditions and the parasitoid’s habitat, *T. triozae* parasite levels can range between 5 and 95% [19,20,21]. *T. triozae* parasitism toward *B. cockerelli* nymphs is regularly affected by multiple factors, such as the lack of alternate hosts, the asynchrony of the host and parasitoid, the size of the sown fields that limits the dispersal capacity, the synthetic insecticides’ application, and the presence of hyperparasitoids [20,22]. Furthermore, the population of parasitoids is also influenced by the domestication level of the host plant, which emits a blend of volatile organic compounds and affects parasitism and parasitoid feeding behavior [23]. The emission of these volatile compounds can help natural enemies locate their prey effectively [24]. Thus, plant volatiles depending upon the type of host plant species or cultivar can significantly influence parasitoid performance in many ways, affecting the parasitism proportion, feeding, survival, female proportion, and longevity of their offspring [25]. Therefore, the objectives of this study were to evaluate the parasitic behavior of *T. triozae* on *B. cockerelli* nymphs in Floradade, Micro-Tom, and Wild tomato varieties and observe their attraction in healthy and infested plants of these varieties.

## 2. Materials and Methods

### 2.1. Plant Growth and Insect Colony

The present work was carried out in the municipality of Saltillo, Coahuila, México, within the facilities of Centro de Investigación en Química Aplicada (CIQA) under low-tech greenhouse conditions (dimensions of 17 × 8 × 4 m and with anti-aphid mesh). The seeds of three tomato varieties were provided by Dr. José Humberto Valenzuela Soto of the Bioscience and Agrotechnology Department of CIQA. Once the seeds were germinated, the 25-day-old seedlings were transplanted in plastic pots with a 1.5 L capacity with peat moss and perlite mixture in a 2:1 ratio and were watered three times a week with Steiner nutrient solution at 25% [26]. Subsequently, 15 days after transplantation, they were used for bioassays. The plants were kept under greenhouse conditions at 28 ± 2 °C and RH = 45%.

The *B. cockerelli* colony was provided by the Molecular Parasitology Laboratory of Parasitology Department from Universidad Agraria Autónoma Antonio Narro. The *B. cockerelli* adults were reared in potato plants (*Solanum tuberosum* L.), Agata variety. The psyllid colony was kept under greenhouse conditions at 28 ± 2 °C in a 60 × 80 × 80 cm wooden cage with an aphid-proof mesh. The parasitoids of *Tamarixia triozae* were acquired from Koppert Biological Systems—Mexico company, located in the state of Querétaro, México. The stage of the parasitoids was 0–7 days.

### 2.2. Parasitism Preference Assay

In a greenhouse, three plants of Floradade, Micro-Tom, and Wild tomato varieties were placed inside a 50 × 50 × 50 cm wooden cage covered with organza fabric. In each cage, 250 non-sexed adults of *B. cockerelli* were released for 72 h; then, the insects were removed, and the eggs placed on each plant were counted. This assay was replicated twice. The eggs hatched, and development was allowed until the nymphs passed to the third nymphal stage (N3); at this time, *T. triozae* adults were released for 24 h to parasitize the nymphs, then *T. triozae* adults were removed. After 14 days, the *T. triozae* adults’ emergences in parasitized nymphs on each plant used in the assay were counted [21,27].

### 2.3. Olfactometer Assay

Y-shaped glass tube olfactometers were used to determine the *T. triozae* attraction to infested (with *B. cockerelli* N3 nymphs) and non-infested plants belonging to either of the three tomato varieties. The olfactometers were 1.3 cm in diameter, 12 cm in length from the base of the tube to the union of the “Y” arms, and arms were 13 cm in length (angle of 45°) [28,29,30]. The first part of the assay consisted of using plants of three varieties infested with nymphs N3 of *B. cockerelli* and placing each of them in a respective desiccator adapted with an air source so that this would drag the aromas. An activated charcoal filter was placed between the air source and the desiccator to remove impurities from the air. The air was allowed to circulate for 10 min before introducing the respective plant into the desiccator. Five minutes after placing the plants, 40 parasitoids were placed at the end where the desiccator air exited. Their behavior was observed for 10 min. When *T. triozae* adults crossed two centimeters from the arm of the Y-tube, it was taken as attraction. Three repetitions were performed separately, using 100 parasitoids in total. The plant combinations were: Floradade vs. Wild, Floradade vs. Micro-Tom, and Wild vs. Micro-Tom. The second part of the essay placed plants of the same variety infested and free of *B. cockerelli* nymphs in the desiccator. The procedure was the same as mentioned above. The assay was performed for each variety. 

### 2.4. Statistical Analysis

The experiments were established under a completely random design, and the data collected were transformed by the arcsine square root transformation and were later used to perform an ANOVA. When necessary, the comparison between means was made using a Tukey multiple range test (*p* = 0.05). Statistical analyses were made using the statistical package SAS version 9.0.

## 3. Results

### 3.1. Parasitism Preference Assay

**Figure 1:**
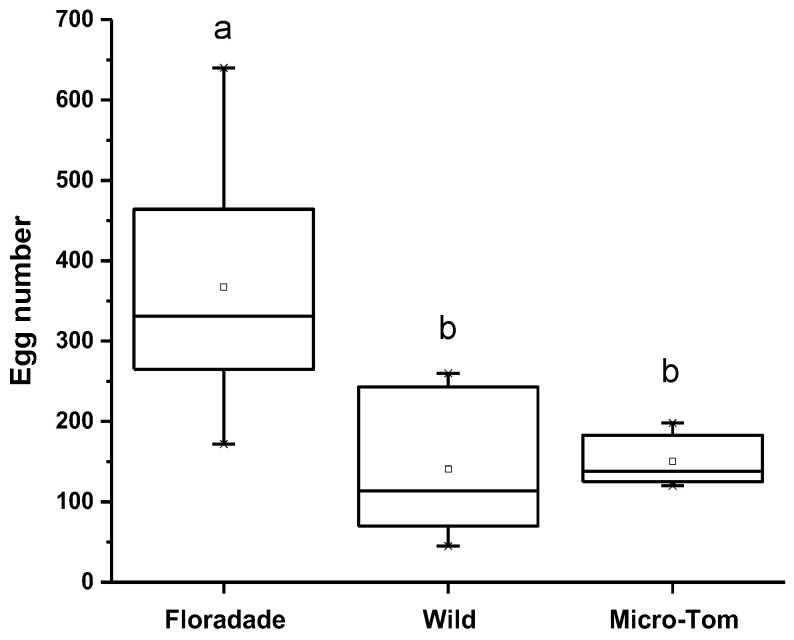
The number of eggs of *B. cockerelli* in tomato plants of three varieties (Floradade, Wild, and Micro-Tom). Different letters indicate statistical differences (*p <* 0.05).

When *B. cockerelli* was released on three tomato varieties, they had the option to oviposit. The oviposition preference was highest on Floradade (domesticated variety) with 367 eggs per plant; meanwhile, 150 and 141 eggs per plant were present on Micro-Tom and Wild, respectively. The statistical analysis showed a significant difference (*p* = 0.0046) for the Floradade variety compared to Wild and Micro-Tom. In contrast, between Wild and Micro-Tom varieties, no significant differences were detected (Figure 1).

**Figure 2:**
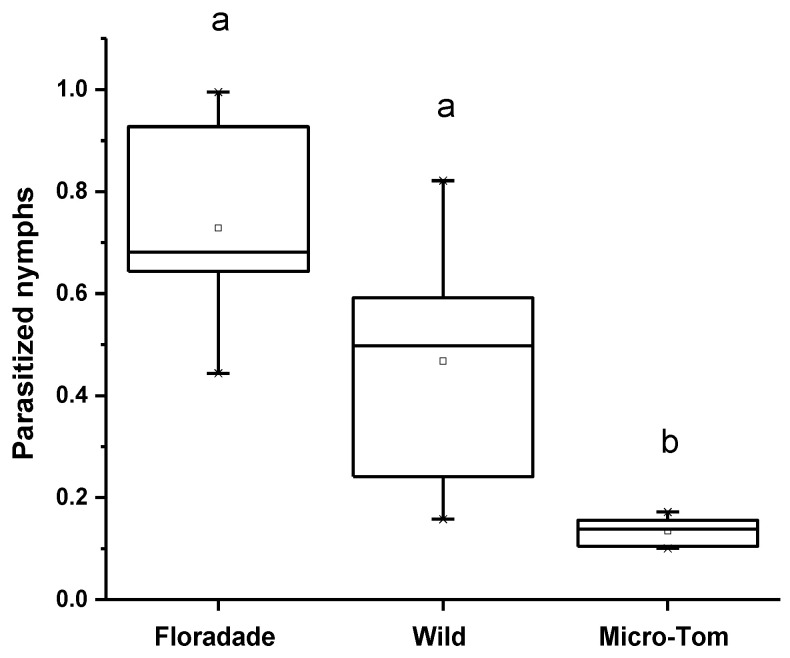
Average nymphs of *B. cockerelli* parasitized by *T. triozae* in different tomato varieties (Floradade, Wild, and Micro-Tom). Different letters indicate statistical differences (*p <* 0.05).

The proportion of parasitized N3 nymphs was different among the three varieties for the parasitism assay. The parasitism percentage in Floradade was 44.6%, followed by Wild and Micro-Tom, 23.1% and 1.8%, respectively. The *T. triozae* performance was more remarkable in domesticated plants than in the Wild and Micro-Tom, plants with a lower domestication degree (Figure 2). The emergence of *T. triozae* adults in Floradade plants occurred 11 days after the parasitoid was removed, while in Wild and Micro-Tom varieties, the emergence was recorded after 14 days. The statistical analysis showed a significant difference in parasitism among the different varieties (*p* = 0.0002).

### 3.2. Olfactometer Assay

**Figure 3:**
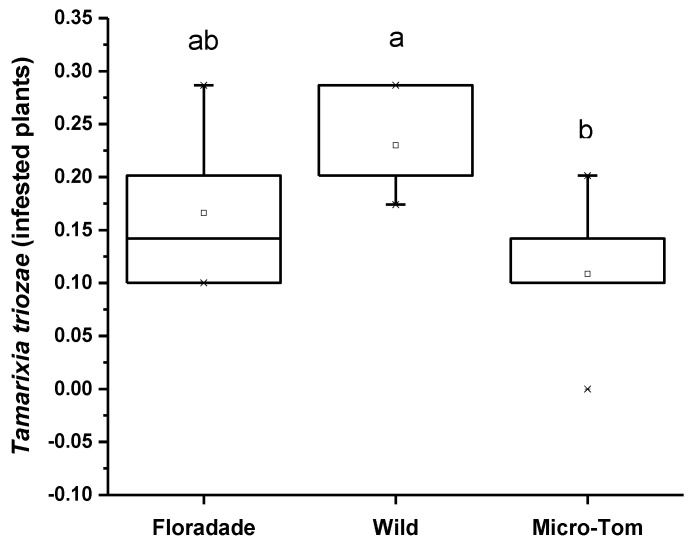
Average number of *T. triozae* adults attracted toward infested plants with N3 nymphs of *B. cockerelli* in different tomato varieties (Floradade, Wild, and Micro-Tom). Different letters indicate statistical differences (*p <* 0.05).

The attraction preference in the Y-tube of *T. triozae* was evaluated in plants of different varieties infested with nymphs of *B. cockerelli*. The Wild variety was significantly more attractive for the parasitoid with a mean of 0.23, followed by the most domesticated varieties, Floradade and Micro-Tom, with an average of 0.17 and 0.10, respectively (Figure 3). The statistical analysis shows a significant difference (*p* = 0.0523) in the three varieties used.

**Figure 4:**
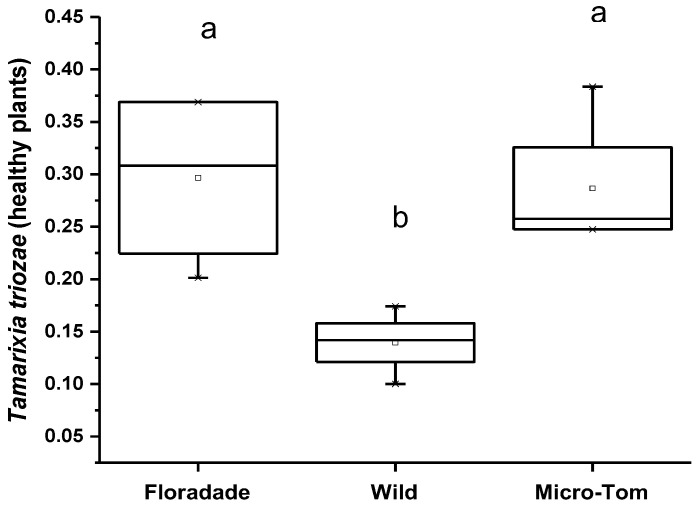
Average number of *T. triozae* adults attracted to *B. cockerelli* nymph-free plants of different tomato varieties (Floradade, Wild, and Micro-Tom). Different letters indicate statistical differences (*p* < 0.05).

Olfactometer tests (Y-tube) showed that N3-free plants were more attractive than plants infested with nymphs, except in plants of the Wild variety, where the damaged plants attract more parasitoids. In healthy plants (free of nymphs of *B. cockerelli*), *T. triozae* attraction was more significant in Floradade and Micro-Tom varieties with an average of 0.3 and 0.29; meanwhile, the Wild variety showed minor attraction for *T. triozae* adults with an average of 0.14, respectively (Figure 4). The statistical analysis showed no significant difference between Floradade and Micro-Tom varieties. However, both varieties attracted significantly higher parasitoids than the Wild variety (*p* = 0.0127).

## 4. Discussion

This study found that the highly domesticated tomato variety, Floradade, had more oviposition of *B. cockerelli* than the less-domesticated ones, Wild and Micro-Tom. The number of eggs deposited in different varieties may be due to factors of the plant itself, such as plant size, leaf color, glandular trichomes presence, nutritional quality, and volatile compounds released; these factors affect the insect’s decision to oviposit [31]. Previous studies reported that the Wild variety presented a reduced preference for *B. cockerelli*; they also speculated that volatiles’ emission in Wild plants could act as a repellent to *B. cockerelli* [32].

The volatile compounds production by domesticated plants is different from Wild plants’ compounds [24,32], which implies a greater or lesser attraction to insect pests. *Tamarixia triozae* parasitism had significant differences in the three varieties. Floradade had the highest percentage of parasitized N3 nymphs, and the lowest percentage was observed on Micro-Tom. The parasitism percentage in the Wild variety was intermediate compared to the other two varieties. These results differ from earlier reports [24] that reported that *Cotesia congregate* (Say), the parasitoid of *Manduca sexta* (Linnaeus), was more attracted to Wild tomato plants than domesticated tomato plants. They attribute this preference to the release of different volatile compounds emitted by plants. Hernández-Moreno et al. [25] found that in tritrophic interactions, the first level affects the performance of the third trophic level by showing that the *T. triozae* population, which was reared on chili, provided 3% more parasitism on chili plants than on tomato. These same authors mention that better understanding the interaction of the Solanaceae–*Bactericera cockerelli*–*Tamarixia triozae* model can lead to optimized control of the pest via taking advantage of positive influences on parasitoid performance. Similarly, Salas-Araiza et al. [33] found that *B. cockerelli* parasitism by *T. triozae* was higher in jalapeño pepper plants than in potato and tomato plants, thus demonstrating that the host affects the third trophic level.

Regarding emergence time, adults of *T. triozae* in the Floradade variety emerged at 11 days, parasitizing the N3 nymphs, while in Wild and Micro-Tom varieties, the emergence of adults was after 14 days. Early emergence in Floradade plants can increase the parasite population quickly within the agro-ecosystem. This could be very helpful in Integrated Pest Management. An essential factor contributing to early emergence might be the availability of nutrients in *B. cockerelli* nymphs, which accelerates parasitoid growth and development. In laboratory conditions, it was found that adults of *T. triozae* emerged after 12 days of oviposition in the fourth and fifth instar nymphs of *B. cockerelli* feeding on tomato plants [16]. When *T. triozae* preference was directly compared in damaged plants of the three varieties, a more significant proportion of the parasitoids chose Wild plants instead of domesticated ones (Floradade and Micro-Tom). The volatile compound’s production is an essential mediator in the attraction of parasitoids and pests [24]. Furthermore, the volatile compound’s production in domesticated plants is different from that of Wild plants and has an ecologically important implication in attracting natural enemies of the pests. In the olfactometer test (Y-tube), where *T. triozae* and infested plants were used versus N3-free plants, the parasitoids significantly preferred N3-free plants over the plants infested with N3 nymphs, except in plants of the Wild variety, where the parasitoids preferred infested plants more than N3-free plants. Thus, our results show that the varieties’ domestication in Floradade and Micro-Tom could have positively influenced *T. triozae* attraction before the arrival of *B. cockerelli*, which is contrary to the previous report by Li et al. [24]. The parasitoid attraction to damaged plants of the Wild variety could be attributed to volatile compounds’ emission, which is responsible for recruiting *T. triozae* when *B. cockerelli* attacks the plant. The domestication degree in plants does not always negatively influence the natural enemy’s attraction.

## 5. Conclusions

Regarding tomato varieties used in this work, it was found that the Floradade variety presented the highest percentage of parasitized nymphs. In addition, it was demonstrated that the domestication of Floradade alerted the plant biochemistry in a way that it interacts positively with the natural enemy of *B. cockerelli.* Therefore, this variety’s cultivation and combination with the release of *T. triozae* is recommended when *B. cockerelli* infests crops.

## Data Availability

The data obtained during the bioassays are available from the corresponding author on reasonable request.
